# Systematic Review on Internet Support Groups (ISGs) and Depression (2): What Is Known About Depression ISGs?

**DOI:** 10.2196/jmir.1303

**Published:** 2009-09-30

**Authors:** Kathleen M Griffiths, Alison L Calear, Michelle Banfield, Ada Tam

**Affiliations:** ^1^Centre for Mental Health ResearchThe Australian National UniversityCanberraAustralia

**Keywords:** Depression, consumer participation, Internet, self-help groups

## Abstract

**Background:**

Internet support groups (ISGs) are a popular means by which consumers with depression communicate online. A number of studies have evaluated the nature and impact of depression-specific ISGs. However, to date there have been no published systematic reviews of this evidence.

**Objective:**

The aim was to systematically identify and summarize the available evidence concerning the scope and findings of studies of depression ISGs.

**Methods:**

Three databases (PubMed, PsycINFO, Cochrane) were searched using over 150 search terms extracted from relevant papers, abstracts, and a thesaurus. Papers were included if they employed an online peer-to-peer depression-specific support group and reported either quantitative or qualitative empirical data. The objective of each study was coded according to a 20-category classification system, which included the effect on depression and other outcomes, including help seeking; user characteristics, activity, satisfaction, perceived benefits, perceived disadvantages; the reason for using the ISG; the nature of ISG posts; characteristics of depression ISGs compared to other ISG types, face-to-face groups, and face-to-face counseling; ISG structure and longitudinal changes; and predictors of ISG adherence.

**Results:**

Thirteen papers satisfied the inclusion criteria from an initial pool of 12,692 abstracts. Of these, three collected data using survey questionnaires, nine analyzed samples of posts, and one both collected survey data and analyzed a sample of posts. The quality of most studies was not high, and little data were collected on most key aspects of depression ISGs. The most common objective of the studies was to analyze the nature of the posts (eight studies) and to describe site usage (six studies) and user characteristics (five studies). The most prevalent types of social support were emotional, informational, and social companionship.

**Conclusions:**

Given the popularity of depression ISGs and the paucity of available evidence about them, there is a need for high-quality, systematic studies of these groups, their impact, and the characteristics of their members and users. Such information is required to inform decision making by consumers, provider and educational organizations, guideline developers, policy makers, and funding bodies considering using, recommending, providing, or funding such groups*.*

## Introduction

Depression is a recurring, debilitating condition that is the primary cause of disability burden in developed countries [1]. Although frequently chronic in nature [2], depression is typically managed as an acute condition. In addition, depression and help seeking for the condition are stigmatized [3,4]. A substantial minority of people with depression do not seek formal help [5], and those who do may find that formal services do not meet all their needs [6], particularly in relation to practical advice and ongoing emotional support. Moreover, it has been calculated that optimal treatment using the best currently available evidence-based interventions would avert only 34% of the burden associated with depression even if it were provided to all people with the condition [7].

It is perhaps not surprising then that consumers seek less-formal methods to assist them in coping with depression. Peer-to-peer depression Internet support groups (ISGs) provide one potential means of obtaining such support. In fact, there is evidence that such groups are among the most common support groups on the Internet [8]. Many ISGs enjoy a large membership. For example, at the time of writing, one depression group reported a registered membership of over 30,000 members with as many as 3993 visiting on one day [9].

Given the prevalence of depression ISGs and their potential to play a role in the management of depressive disorders, it is important to understand who, why, and in what way consumers use depression ISGs, their benefits and risks, and how such groups are best structured for optimal consumer outcomes. In a companion paper, we have reported the results of a review of the effect of health ISGs on depressive symptoms [10]. However, the review was not focused on depression ISGs specifically and incorporated only those studies reporting depression outcome data. As noted above, other attributes of depression ISGs are of interest. To our knowledge, there are no published reviews of the scope or findings of empirical research on depression-specific ISGs.

The current study seeks to address this gap by reporting the results of a systematic review of the available quantitative and qualitative evidence concerning depression ISGs. In particular, the review aims to document what is known about the demographic and clinical characteristics of depression ISG users, the nature and quantity of depression ISG usage, consumer attitudes about depression ISGs, whether depression ISGs influence help seeking and user attitudes about conventional health care, and how these online groups compare with other types of ISGs and with face-to-face mutual support groups for depression.

## Methods

The methods employed in the current review have been reported in a companion study of the efficacy of health ISGs in reducing depression symptoms, [10] and the reader is referred to that paper for further details. Briefly, the review methodology entailed a search of three databases for the period prior to August 2007 using an extended version of a search strategy reported by Eysenbach et al [11]. The procedure for identifying studies involved a multi-step process: (1) eliminating clearly irrelevant abstracts, (2) identifying definitely or possibly relevant abstracts (two reviewers), (3) collecting and eliminating papers not satisfying inclusion criteria (two reviewers), and (4) identifying any additional relevant studies cited in systematic reviews (two reviewers) or included studies. Inclusion criteria for the current study were that the study reported qualitative or quantitative empirical data on a peer-to-peer depression support group. Studies were excluded if the target ISG was not specific to depression [12-14]. Of the 12,692 abstracts initially returned by the database searches, 13 studies satisfied the inclusion criteria, including two efficacy studies [15,16] that were also included in the efficacy review [10]. A flow diagram of the above process is available in the companion paper to this review [10]. As noted by Griffiths et al [10], it is possible that additional papers may have been published since the original searches. To investigate this, a search was conducted by the first author incorporating the period of 2007 to May 2009 and using the same search terms employed in the reported searches but limiting results to those incorporating the terms “depression” or “depressive” or “mood.” No new empirical studies of depression ISGs were identified.

### Coding of the Included Studies

Each of the identified 13 papers was independently coded by two raters (KG, AT) and discrepancies subsequently resolved by discussion. Variables coded included type of ISG (format, moderation characteristics, country of origin), country of origin of the primary author, study design (experimental, observational, descriptive), and the type of data analyzed (survey, ISG posts). In addition, each study was rated for the presence (yes, no) of each of a series of potential aims: to measure the (1) effectiveness of ISGs on depression or (2) another outcome (eg, quality of life); (3) effect on formal service use; (4) user satisfaction with depression ISGs; (5) reason for using depression ISGs; (6) user perceived benefits; (7) user perceived disadvantages; (8) source of referral to the ISG; (9) demographic characteristics; (10) clinical characteristics; (11) service use of depression ISG users; (12) activity/usage of depression ISGs; (13) the nature of depression ISG posts; (14) comparative characteristics of depression and other medical ISGs, (15) face-to-face support groups, and (16) face-to-face counseling; (17) changes in depression ISGs over time; (18) predictors of adherence to depression ISGs; (19) aspects liked best and/or least about ISG; and (20) ISG structure. In practice, it proved difficult to differentiate between categories (5) and (6), and these were therefore combined into a single category, “reason for using depression ISGs and/or user perceived benefits.”

## Results

Table 1 summarizes the characteristics of each of the 13 identified studies. The majority were descriptive (n = 11), one was an observational trial, and one was an experimental trial. The quality of most studies was not high, and little data had been collected on most key aspects of depression ISGs. The most common objective of the studies was to analyze the nature of the posts (eight studies) and to describe site usage (six studies) and user characteristics (five studies). Two of the studies analyzed different aspects of the same data [17,18]. Findings from the 13 studies of depression ISGs are detailed below.

**Table 1:** Design characteristics of depression ISG studies: ISG user survey studies, ISG post studies, and combined user and post studies^a,b^

Study	Setting/Type of ISG	Design	Recruitment (surveys)/ Sampling method (posts)	Aims (for which findings reported)	Measures
**ISG user survey studies**					
Houston 2002 [15] USA	Five moderated public depression bulletin boards and listservs	Observational (survey) Descriptive (survey)	Participants recruited for survey over 2-mth period through posts on the five ISGs	Effectiveness depression Effectiveness other outcome Characteristics Perceived benefits/ disadvantages Activity/usage Compare with face-to-face counseling	CES-D (depression) MOS-SS (social support) Age, gender, marital status, education, employment, nationality, years since first diagnosis, type/quantity/quality face-to-face care Value of ISG (5-point Likert), effect on face-to-face care ISG use past 2 wks (hrs), self-report Preference for type of interaction (face-to-face vs ISG)
Powell 2003 [19] UK	Six Netdoktor depression bulletin boards (Austria, Denmark, Germany, Norway, Sweden, UK) Moderation status N/S	Descriptive (survey)	Visitors to Netdoktor ISGs over 4-wk period in May/June 2002 responding to survey offered in pop-up window	Characteristics Perceived benefits/disadvantages	Age, sex, history of depression/consultations, MDI, nationality of ISG, reason for visit (self, friend, family member, etc) Self-perceived effects of use
Andersson 2005 [16] Sweden	Moderated, research depression bulletin board	Experimental Control arm of a randomized controlled trial (survey)	Recruitment of participants for an Internet research project on depression through print media Self-selected participants included if probability of 0.55 of diagnosis of MDD (CIDI-SF) and clinically significant symptoms of depression (MADRS-S)	Efficacy depression Efficacy other outcome Predictor of adherence	BDI, MADRS-S (depression) QOLi (quality of life), BAI (anxiety)
**ISG post studies**					
Davison 2000 [8] USA	Highest volume English-language Internet depression newsgroup and AOL depression support bulletin board	Descriptive (posts)	All posts to ISG analyzed over 2-wk period	Activity/usage Compare ISGs	Number of posts
Salem 1997 [20] USA	Public depression newsgroup	Descriptive (posts, 2 independent coders)	Analyzed all posts to ISG in each of 2 wks, 1 mth apart in 1995 (involving 533 posters)	Characteristics Activity/usage Nature of posts Compare with face-to-face group	Gender, work status, whether depression professionals (inferred from posts); whether depressed or a carer (inferred from posts) Frequency of posts, no. of posters, mean and range posts/person, target of post (group vs individual) 13 coding categories (5 general categories) derived from Roberts [21] and face-to-face mutual support literature Measures used for characteristics and nature of posts
Fekete 2002 [22] Hungary	Depression newsgroup	Descriptive (posts)	Analyzed all posts to ISG over 3-mth period (involving n = 45 posters)	Characteristics Activity/usage Nature Compare ISGs	Gender (inferred from posts), nationality (inferred) Frequency of posts, no. posters, no posting once only Syntax/grammar analysis/speech patterns/verbal features (modified Weingraub [23,24] content analytic method)
Muncer 2000 [17] UK	One depression newsgroup, selected because it was “particularly active”	Descriptive (posts)	Random sample of all postings to ISG made over 1 mth (involved n = 118 participants)	Usage Nature	Mean thread length, no posting > 6 messages Cohen and Wills [25] typology of social support (coded at level of thread)
Muncer 2000 [18] UK	Depression newsgroup from Muncer et al [17] Diabetes newsgroup	Descriptive (posts)	Analyzed posts made over 1 mth to depression ISG (sampled as above) and posts made over “longer period” to diabetes ISG Usage analyses based on all posters (n = 118 depression, n = 132 diabetes); network structure on frequent posters (n = 26 depression, n = 10 diabetes)	Activity/usage Network structure Compare ISGs	Mean thread length, posters/thread, posts/person, no. posting once only, no. posting > 6 messages Multidimensional scaling routine form UCINET [26]
Alexander 2002 [27] USA	Three public depression ISGs selected randomly from a list of non-professionally run depression groups on public e-groups website Moderation status N/S	Descriptive (posts, partial double coding to establish and verify reliability)	Analyzed 1 mth of posts to the three ISGs at 3, 6, 9, and 12 mths after group commencement Posts from 1998 to 2001	Activity/usage Nature (for leaders/most active participants)	Frequency of posts/mth, no. posters, no. leaders Modified Cutrona’s Support Behaviour Codes [28]
Witt 1999 [29] USA	Public depression bulletin board, public smoking cessation bulletin board Method of selection, moderation status N/S	Descriptive	Analyzed over 1000 posts on ISGs collected over a “2- to 3-mth period”	Nature of posts Comparison of ISG types	Contract, Thinking Feeling (CTF) coding system [30]
Macius 2005 [31] UK	Public “message board”	Descriptive (posts, 3 coders, reliabilities computed)	Analyzed one or two threads on ISG for a week in June 2002	Nature of posts Comparison of ISG types	Coded for type of support, type of medical/drug content discussed
Lamerichs 2003 [32] Netherlands	Public depression “discussion forum” (senior citizens)	Descriptive (posts)	Selected extracts from ISG, chosen to illustrate limitations of existing cognitive models of online interactions	Nature of posts	Identity management
**Survey and posts**					
Alexander 2003 [33] USA	Newsgroup Method of selection and moderation status N/S	Descriptive (posts, 3 independent coders)	Posts: Analyzed all posts made to the ISG over 3 consecutive wks (n = 74 members posting) Survey: Volunteers recruited through post on the ISG (n = 19)	Characteristics Usage Nature	Depression status (disclosed in posts) Frequency of posts, no. of posters Cutrona’s Support Behaviour Codes [28] 6 satisfaction items (survey)

^a^ AOL = America Online; BAI = Beck Anxiety Inventory; BDI = Beck Depression Inventory; CES-D = Center for Epidemiologic Studies Depression Scale; CIDI-SF = Composite International Diagnostic Interview Short-Form; MADRS-S = Montgomery-Asberg Depression Rating Scale; MDI = Major Depression Inventory; MDD = Major Depressive Disorder; MOS-SS = Medical Outcomes Study Social Support Scale; N/S = not specified; QOLi = quality of life.

^b^ Only measures for which data or findings were reported are included in table.

### What Are the Characteristics of Depression ISG Users?

There is limited research on the clinical and demographic characteristics of the users of public depression ISGs. Moreover, data collected thus far have been derived only from surveys or advertisements posted on bulletin boards or inferred from bulletin board posts. No study has reported data on characteristics of all users who register with an ISG.

#### Clinical Status

Four studies reported information about users’ current or past history of depression [15,19,20,33]. Assessments employed included formal measures and self-reported status as well as mental health status inferred from the content of ISG posts. The evidence suggests that the majority of depression ISG users are consumers with depression or a history of depression and that a substantial percentage (50-80%) are currently depressed on formal assessment. In particular, Houston et al [15] found that 99% of respondents to a survey of depression ISG members reported having received a diagnosis of depression from a health professional, and 86% scored above the cutoff for depression on the CES-D screening test for depression at the time of completing the survey 1 to 2 months after joining the ISG. Powell et al [19] reported that 52% of ISG users had current major depression as measured by the Major Depression Inventory and that 7% of respondents were friends or family members of a person with depression. They argued that the discrepancy between their findings and those reported by Houston et al [15] may be due to differences in recruitment methods employed in the two studies. The remaining two studies reported the rates of self-identification or inferred depression status from an analysis of posts [20,33]. The first found that all members posting on a depression ISG self-identified as suffering from depression, [33] and the second, inferring the clinical status of active users of a newsgroup from their posts, reported that 92% identified themselves as depressed and 2% as carers of people with depression [20].

#### Clinical Treatments/Service Use

Two studies have reported data on depression ISG users’ receipt of professional treatments or services [15,19]. Houston et al [15] reported a high level of such help, with 92% of ISG respondents to their survey currently receiving antidepressants and 65% receiving counseling. ISG users surveyed by Powell et al [19] were less likely to have received professional help. Reporting only on the 50% of depression ISG participants with current major depression, the authors found that 26% were currently receiving psychological treatment, 44% medication for the condition, and 51% either psychological treatment, medication, or both. Although 64% had consulted a health professional in the previous year, one-third (34%) had never received either treatment. Again, it is unclear if the discrepancy in the results of these studies relates to differences in the study recruitment methods or to differential treatment rates in the jurisdictions served by the ISGs [19].

#### Gender

Four studies analyzed the gender distribution of users [15,19,20,22]. The results of these studies were mixed. Two reported a preponderance of females (79% and 70%) [15,19], and two reported more male users (61% and 66%) [20,22]. The first two studies focused on bulletin boards, and gender was based on self-report among survey respondents; the second two studies involved newsgroups, and gender was inferred from posts.

#### Age

Two studies reported on the age distribution of respondents. These studies suggest that bulletin board users are commonly between their mid- to late 20s and mid-40s. Houston et al [15] reported that 21%, 49%, and 30% of bulletin board participants were aged 18-29 years, 30-45 years, and over 45 years, respectively. Powell et al [19] reported that 27%, 33%, 23%, and 17% of participants were aged < 26 years, 26-35 years, 36-45 years, and over 45 years, respectively.

#### Other Demographics

Only one study evaluated other demographic characteristics of depression ISG users. Houston et al [15] reported that 45% of users had achieved a college education, 42% were unemployed, and 44% were married.

#### Source of Referral

Although one survey included a question about source of referral to the depression ISG, the authors did not report the findings for this item [33].

### What Is Known About Depression ISG Usage?

Six studies (seven papers) provided some information about usage of public depression ISGs [8,15,17,18,20,22,27].

#### Posting Rate

Three studies reported the rate of posting on an ISG, with markedly differing results. For comparative purposes, we converted these rates to posts per user per week. Reported rates of posting varied from 0.3 [22] to 25.4 [27] per user per week. More specifically, in studies of depression newsgroup posts, Salem et al [20] reported 1.8 posts per user per week (533 users over 2 weeks) and Fekete [22], 0.29 posts per user per week (45 users over a 3-month period, 29 [64%] of these posting only once). Alexander [27] found that the number of users and rate of posting on a depression ISG varied across time and group. Twelve months after commencement of the group, usage on three different depression ISGs was 5.6 (20 participants), 25.4 (21 participants), and 1.2 (69 participants) posts per user per week. Usage of the ISGs also changed over time (see below). Davison [8] reported that the highest-volume depression ISGs on America Online and the wider Internet yielded total weekly posts of 124 and 389, respectively. However, the author failed to report the number of users generating these posts.

#### Thread Length

Little is known about average thread length on depression ISGs. One group reported an average thread length of 8.04 posts across 61 threads randomly selected from an unspecified number of threads posted during a 1-month period on a “particularly active newsgroup,” with 60% of messages posted by 26 (22%) of the participants [17].

#### Time Spent on ISGs

Only one study investigated the time members devote to depression ISGs. Over half (53.4%) of the ISG users in the study reported spending at least 5 hours on the depression ISG over a 2-week period in the early stages of their membership in the group [15].

### What Is the Nature of Posts on Depression ISGs?

Eight studies (nine papers) provided some information about the nature of depression ISG posts [17,18,20,22,27,29,31-33]. Four of these reported quantitative information about the prevalence of different types of social support in posts on a total of seven newsgroups [17,20,27,33]. Some of the latter studies also coded for other characteristics, including the prevalence of disclosure, help seeking, different types of knowledge [20], and task maintenance [17,33].

Each employed a pre-formulated system for coding the typology of posts. Coding systems included the Cutrona Support Behavior Code [28] (studies [27,33]), an adaptation of a typology developed by Roberts and collaborators for face-to-face support groups [21] (studies [20,21]), and a typology of social support described by Cohen and Wills [25] (studies [17,27]). Each of these systems differs subtly, rendering synthesis of study findings difficult. For example, cognitive guidance messages were coded as informational in some studies [17,27,33] but separately in another [20]. Esteem support and emotional support were treated separately in two studies [27,33] but coded only in the category “esteem support” in another [17]. Studies also differed with respect to the unit of analysis (thread vs post) and whether the unit could be allocated a single code or multiple codes. One study confined the analysis to messages that the authors deemed “helping posts” [20], and another limited the analysis to a very small number of members designated as group “leaders” [27]. The characteristics of the depression ISGs also differed across and within studies.

#### Social Support

The relative prevalence of different types of social support varied across studies. However, overall emotional support was common (mean 34%, range 22-48%, n = 5 groups). Informational support also accounted for a substantial proportion of posts (mean 26.2, range 8-46%, n = 6 groups). In the single study that investigated it [20], cognitive guidance accounted for only a minority of posts (7.2%). Posts sharing experiential knowledge (14%) were more common than posts containing “second-hand” professional knowledge (3%) [20]. The only study that reported prevalence of social companionship (eg, social chit-chat) found that it accounted for almost one quarter of posts [17]. Considering only the two studies that coded pure self-esteem, one reported a substantial minority of self-esteem posts (24%, [33]), whereas the other, which was confined to posts of ISG leaders, reported few self-esteem posts (5%, [27]). Unlike other forms of social support, tangible support was absent in ISG posts [17,33].

#### Other Characteristics of Posts

Salem et al [20] reported that self-disclosure was common (50.6%) but that only a minority of posts involved requests for help (13%). Posts relating to the group structure and group identification were also common (20%). However, in his study of three depression ISG groups, Alexander [27] identified relatively low levels of task maintenance (eg, monitoring group norms, keeping discussion on track; mean 5%, range 2-8%, n = 3 groups) and relational maintenance (reinforcement of the cohesiveness of the group; mean 9.3%, range 8-11%, n = 3).

In a syntactical and grammatical analysis of ISG posts, another study reported that personal “I/me” references, feelings, expressions involving judgments of goodness/badness, adverbial intensifiers, dichotomous expressions, retractions and explanations were more frequently employed on a depression ISG than a control group journalism discussion group [22].

### What Predicts the Nature of Posts?

Salem et al [20] found that high-frequency users were significantly more likely to post socially supportive and humorous messages, to agree with others, and to respond to individuals as opposed to the group. However, they disclosed less, sought less help, and offered less experiential and second-hand knowledge than less-frequent ISG users. Salem et al [20] also investigated difference in posts according to the inferred gender of the user. A larger percentage of men’s posts were experiential, whereas women’s posts were more likely to involve group structure and process.

### Are Users Satisfied With Depression ISGs?

Only one study has formally measured level of satisfaction with a depression ISG [33]. Based on a survey posted on a depression ISG, the authors reported that members were extremely satisfied with the group (score 6.47 out of 7). The response rate represented 26% of 74 members posting during the period the survey was available.

### Why Do Members Use ISGs?: Perceived Benefits and Disadvantages

Three studies systematically investigated the nature of benefits and/or the reason for participating in a depression ISG [15,19,33]. They provide some evidence that emotional support and information are perceived by members as an advantage of depression ISGs and that these groups are perceived to be effective in reducing depressive symptoms. There is little evidence concerning the disadvantages of ISGs.

In particular, Houston et al [15] reported that emotional support was the most commonly cited reason for participating in a depression ISG. Of the 103 members who participated in their survey, 98 (95%) reported that the ISG had helped their symptoms. Powell et al [19] found that of those members who visited a depression ISG more than once and completed an online survey, the majority (71%) reported having learned “more about medication,” half indicated that they were “able to discuss subjects that they felt unable to discuss elsewhere,” and 44% indicated that they “felt less isolated” due to their participation in the ISG [19]. There were also positive effects on formal help seeking (see below). Finally, Alexander et al [33] reported that user-cited benefits of a depression ISG were freely given support, caring, and affirmation from other members, the provision of an outlet for expression, and a place to turn when alone. Although the authors requested that users also indicate the aspects of the depression ISG they liked least, users typically replied that the groups did not require any improvement.

### Do Depression ISGs Improve Outcomes?

Two studies have investigated the effectiveness of depression ISGs for improving outcomes [15,16], in particular for depression [15,16], anxiety [16], quality of life [15], and social support [15]. As noted by Griffiths et al [10], the studies employed different designs, recruitment procedures, and ISG types and yielded different findings for depression, with one reporting a decrease in depressive symptoms among frequent public ISG users relative to low-frequency users and the other showing no significant reduction in depressive symptoms following participation in a research ISG. The only study to examine the effect of an ISG on anxiety symptoms yielded no change following participation in a research ISG [16]. The latter study also failed to find a change in quality of life following participation in the research ISG. There was no effect of participation on social support [15].

There were no observational or experimental studies of the effect of depression ISG participation on other outcomes such as knowledge of depression, attitudes to depression, self-efficacy, self-esteem, behaviors, or user empowerment.

### Does Participation in Depression ISGs Affect Formal Help Seeking?

Little is known about the effect of ISGs on the use of other health services. However, Powell et al [19] found that over one-third (37%) of participants in their survey reported that participation had encouraged them to seek professional help, although a small minority considered that their participation had delayed such a consultation (9%) or reduced their trust in their doctor (11%) [19].

### What Are the Similarities and Differences Between Face-to-Face Support Groups and ISGs?

No study has directly compared face-to-face and online support groups in a single trial. However, one group [20] did compare their findings from a depression ISG with those from a previously reported study of a face-to-face group [21].

#### Type of Interaction

Basing their coding system on that used in the interactions in the face-to-face support group, the researchers concluded that there were similarities and differences between the nature of ISG and face-to-face interactions. In each, “positive, supportive” statements were more frequent than negative comments. However, self-disclosure, which was rarely seen in the face-to-face group, was the most common type of communication in the ISG group. The authors [20] asserted that this difference was unlikely to be due entirely to differences in anonymity in the two formats since many users employed their own identities on the ISG. Rather, they proposed that the absence of physical cues minimizes perceived differences between members and allows participants to focus on the communication and their shared concerns. A second difference between ISG and face-to-face groups was that whereas for ISGs advice and information and emotional support were more common than cognitive guidance (of the type used in conventional therapy), the reverse was true for face-to-face interactions.

#### Demographic and Other Characteristics

The authors of the above study [20] also concluded that the gender composition of ISGs and face-to-face groups differed. They noted that whereas more women than men use face-to-face mutual support groups, 61% of ISG members in their study were male. However, as already noted, they inferred rather than directly collected gender information in their ISG study. It is possible that men were more likely to disclose their gender online.

### Do ISGs for Depression Differ From ISGs for Other Conditions?

Seven studies have provided some comparative information about ISGs for depression and other conditions [8,18,22,27,29,31,33]. These have provided data on the differences in post frequency (n = 1), post content (n = 4), ISG structure (n = 1), and satisfaction with the ISG (n = 1).

#### Activity

Davison [8] investigated the extent of participation (number of posts over a 2-week period) as a function of different types of ISG. Depression ISGs were the third and fourth most active ISGs on America Online and the general Internet, respectively.

#### Content

Some differences have also emerged with respect to the typology of the posts for depression and other online groups.

Alexander et al [33] compared the frequency of types of social support on a depression, Alcoholics Anonymous (AA), attention deficit disorder (ADD), and cancer ISG. Whereas emotional support was the most common type of support provided on the depression ISG, informational support was the most common support on each of the other three ISGs. The extent to which these differences were due to the nature of the condition supported by the ISG as opposed to the smaller membership and greater homogeneity of the depression group is unclear. In another study, Alexander et al [27] compared the type of social support seen on ISGs for eight different conditions, one of which was depression. Three different ISGs were analyzed for each condition. However, it was difficult to draw conclusions about the comparative frequency of different types of support across conditions due to the variability in the results between groups within the same condition. In addition, since only the messages of “leaders” were coded and there were few of these in a number of instances, the generalizability of the results to all members is unclear. In another study of the content of discussions, Macias et al [31] compared the nature of posts on ISGs for depression, panic/anxiety, breast cancer, prostate cancer, infertility, HIV/AIDS, arthritis, type II diabetes, high cholesterol, irritable bowel syndrome, and obesity/overweight groups. They reported that the depression ISG participants were the most likely to seek and provide advice but were among the least likely of the groups to discuss medical treatments and procedures. The depression group did not differ from the other ISGs with respect to seeking encouragement, expressing concern, or providing personal information not related to the illness.

Fekete [22] analyzed the syntactical and grammatical characteristics of a depression, suicide, and panic ISG and a journalism discussion group. Compared to the other three groups, the depression participants were significantly more likely to make value judgments (goodness-badness, right-wrong). They were also significantly more likely than the journalism group to use dichotomous (polarized) expressions such as “always” or “never,” to provide a reason for their thoughts, beliefs, or actions (“because,” “therefore”), to use words retracting another statement (eg, “but,” “on the other hand,” “except”), and to refer to themselves (“I,” “me”). The depression group was more likely to use adverbial intensifiers (“I *really* like it”) and to express feelings (eg, “I love,” “I hate”) than the journalism or panic groups. The suicide group used significantly more negative (“no,” “never”) and dichotomized expressions than did the depression group. In another study that analyzed the terms in a post, Witt [29] compared the content of a depression and smoking ISG using a computerized rating system of four bipolar dimensions: emotion, cognition, contract, and performance (see [30]). She concluded that compared to the smoking group, the depression group’s language incorporated more negative affect (emotion negative) and that the depressive group was more active in asking for help (negative cognition) and offering help and information (positive cognition).

#### Network Analysis

In a comparison of the structure of a depression and a diabetes ISG using network analysis, Muncer et al [18] reported that the depression ISG networks were “denser and more vibrant,” more likely to involve “cliques,” and more likely to be characterized by social support than the diabetes group, in which participants were more likely to exchange information.

#### Satisfaction

Alexander et al ([33], see above) found that participants in the depression ISG reported a higher level of overall satisfaction than did the members of the AA or ADD ISGs. The depression group also reported higher satisfaction with esteem support than did the AA, ADD, or cancer group members.

### How Do Depression ISGs Change Over Time?

Only one study has investigated change associated with ISGs over time [27]. The trend in the pattern of posts over time varied across depression groups, with one steadily decreasing, one increasing, and the third decreasing initially and then leveling out. Similarly, there were no consistent patterns of change across three depression groups in type of social support over a 1-year period.

### What Predicts Adherence to ISGs?

Andersson et al [16] reported a withdrawal rate of 18% over a period of 10 weeks among participants in a stand-alone depression ISG created as part of a research protocol, a figure that was lower than the dropout recorded for an online cognitive behavioral therapy program over the same period. There have been no other reports of adherence rates for depression ISGs and no studies of the predictors of ISG adherence.

### Preferences for ISG Support Compared to Face-to-Face Counseling?

Of those depression ISGs users responding to a survey, 38% indicated that they preferred ISG support to face-to-face counseling, approximately half (51%) preferred counseling, and the remainder expressed no preference [15].

## Discussion

### Findings

The systematic search yielded only 13 relevant studies for the period under study. These studies addressed a range of issues, including efficacy, user characteristics, activity levels, the nature of online interactions, satisfaction, and reasons for visiting depression ISGs. They also compared ISG activity and interactions over time and across conditions. However, the available data on each of these facets of depression ISGs were limited either due to the small number of studies undertaken, to methodological limitations, or both.

For example, little is known about the demographic characteristics of users, and although there have been four studies of gender distribution, results have been mixed and the methodology employed inadequate. Data that have been collected on clinical characteristics of users suggest that a majority of ISG users have a history of depression and that a substantial percentage of those with current depression were receiving concurrent conventional depression treatments. The latter finding suggests that ISGs often serve as an adjunct to, rather than a replacement for, formal help seeking. However, one study did find that one-third of members with a current depressive disorder had never received formal treatment [19]. Together with evidence that many members report that a depression ISG facilitated their help seeking [19], this finding raises the possibility that depression ISGs may be a fertile setting in which to encourage formal help seeking. There are no available data concerning the means by which users are referred to or arrive at ISGs.

Although seven papers provided information about usage of depression ISGs, overall posting ratings varied across studies and there were insufficient studies to draw conclusions about thread length and time spent on depression ISGs. The strongest focus for depression ISG research has been on the nature of posts (eight studies, of which four investigated types of social support). The primary types of social support provided on depression ISGs were emotional, informational, and social companionship. In the one study that coded for it, self-disclosure was high. The effect of self-disclosure, including its potential advantages and disadvantages, has not been explored in the depression ISG domain, although there is evidence that high-frequency users are less likely to disclose [20] and that disclosure is higher on ISGs than in face-to-face support groups. There would also appear to be differences across conditions in the typology of posts and structure of ISGs. However, it is difficult to determine the extent to which variations across conditions in ISG interactions and structure are attributable to differences in the conditions or other factors (eg, size of the group, duration of group). One study reported variations across ISG depression groups in activity and type of interactions over time, with no consistent pattern evident across the groups.

The lack of efficacy studies of depression ISGs has already been discussed in the companion paper to this study [10]. There was a similar gap in evidence concerning the impact of depression ISGs on other well-being and mental health outcomes. Moreover, there were no observational or experimental studies of the effect of these support groups on user knowledge, user behaviors, or user attitudes.

Nevertheless, there is some evidence that users believe depression ISGs are useful. The single study to formally investigate user satisfaction with depression ISGs reported satisfaction to be high both in absolute terms and relative to ISGs for other health conditions. However, member response rate in this study was low, so it is unclear if the results are representative of all members. There is some evidence that depression ISG members perceive emotional support [15,33], information [19], and effectiveness in improving mood [15] as advantages of their depression ISG, but again these results may not be representative of all users. Significantly, there is little evidence concerning the disadvantages of ISGs despite the practical importance of this issue.

It is notable that none of the studies addressed the question of what factors promote greater acceptability of and satisfaction with depression ISGs, retention of members, activity levels, or efficacy of depression ISGs. No studies systematically manipulated variables such as group size, presence or absence of a moderator, level of moderator participation, content of the board rules, or level of ISG accessibility to evaluate the effect of these on process or outcome variables. Moreover, there were no comprehensive user reports addressing these issues or naturalistic comparative studies of groups differing in these attributes. It is notable that despite the potential relevance of depression ISGs to specific target groups such as adolescents, who are high users of social networking technologies, and older people and rural residents, whose access to other forms of peer-to-peer mutual support may be limited, there are no published studies of the use of ISGs by these populations. Without such studies, organizations or individuals planning or providing depression ISG services must do so without the benefit of an evidence-based framework.

### Conclusions

The conclusions from this study are clear. There is a need for high-quality research, both quantitative and qualitative, to investigate all aspects of ISGs as they relate to depression. Currently, the evidence is not of sufficient quality or strength to inform decision making by consumers, ISG providers, health professionals, policy makers, or funding bodies considering the use of ISGs for depression*.* Given the popularity of peer-to-peer depression ISGs, appropriately targeted studies of these mutual support groups has the potential to contribute substantially to the identification, design, and implementation of suitable self-care models for depression.

## Abbreviations

ISG: Internet support group
